# Lysine, Lysine-Rich, Serine, and Serine-Rich Proteins: Link Between Metabolism, Development, and Abiotic Stress Tolerance and the Role of ncRNAs in Their Regulation

**DOI:** 10.3389/fpls.2020.546213

**Published:** 2020-12-03

**Authors:** P. B. Kavi Kishor, Renuka Suravajhala, Guddimalli Rajasheker, Nagaraju Marka, Kondle Kavya Shridhar, Divya Dhulala, Korubothula Prakash Scinthia, Kummari Divya, Madhavi Doma, Sujatha Edupuganti, Prashanth Suravajhala, Rathnagiri Polavarapu

**Affiliations:** ^1^Department of Biotechnology, Vignan’s Foundation for Science, Technology and Research (Deemed to be University), Guntur, India; ^2^Department of Chemistry, Manipal University Jaipur, Jaipur, India; ^3^Department of Genetics, Osmania University, Hyderabad, India; ^4^Biochemistry Division, National Institute of Nutrition-ICMR, Hyderabad, India; ^5^Department of Botany, Osmania University, Hyderabad, India; ^6^Department of Biotechnology and Bioinformatics, Birla Institute of Scientific Research, Jaipur, India; ^7^Genomix CARL, Pulivendula, India

**Keywords:** lysine metabolism, serine metabolism, abiotic stress, plant ontology, stress tolerance

## Abstract

Lysine (Lys) is indispensable nutritionally, and its levels in plants are modulated by both transcriptional and post-transcriptional control during plant ontogeny. Animal glutamate receptor homologs have been detected in plants, which may participate in several plant processes through the Lys catabolic products. Interestingly, a connection between Lys and serotonin metabolism has been established recently in rice. 2-Aminoadipate, a catabolic product of Lys appears to play a critical role between serotonin accumulation and the color of rice endosperm/grain. It has also been shown that expression of some lysine-methylated proteins and genes encoding lysine-methyltransferases (KMTs) are regulated by cadmium even as it is known that Lys biosynthesis and its degradation are modulated by novel mechanisms. Three complex pathways co-exist in plants for serine (Ser) biosynthesis, and the relative preponderance of each pathway in relation to plant development or abiotic stress tolerance are being unfolded slowly. But the phosphorylated pathway of L-Ser biosynthesis (PPSB) appears to play critical roles and is essential in plant metabolism and development. Ser, which participates indirectly in purine and pyrimidine biosynthesis and plays a pivotal role in plant metabolism and signaling. Also, L-Ser has been implicated in plant responses to both biotic and abiotic stresses. A large body of information implicates Lys-rich and serine/arginine-rich (SR) proteins in a very wide array of abiotic stresses. Interestingly, a link exists between Lys-rich K-segment and stress tolerance levels. It is of interest to note that abiotic stresses largely influence the expression patterns of SR proteins and also the alternative splicing (AS) patterns. We have checked if any lncRNAs form a cohort of differentially expressed genes from the publicly available PPSB, sequence read archives of NCBI GenBank. Finally, we discuss the link between Lys and Ser synthesis, catabolism, Lys-proteins, and SR proteins during plant development and their myriad roles in response to abiotic stresses.

## Introduction

Amino acids are organic compounds, which contain amine (-NH_2_) and carboxyl C(=O)OH) functional groups along with a side chain (R group). As amino acids are building blocks of proteins, they participate in the synthesis of hormones in animals and in peptide hormone synthesis in plants (Hirakawa et al., 2017). In the case of plants, amino acids also take part in the synthesis of several secondary plant products. Amino acids that cannot be synthesized by some mammals and humans are generally known as “essential,” and lysine (Lys) is one among them (Bright and Shewry, 1983). The pathway of Lys is always a target for the development of herbicides in addition to increasing nutritional value in cereals. Lys is entangled in histone modifications and, therefore, is associated with epigenome and stress biology in plants (Yuan et al., 2013). Lys is the most limiting in all major cereal grains and is therefore considered as a nutritionally significant amino acid (Wenefrida et al., 2009; Galili and Amir, 2013). This is precisely the reason why Lys is a target for crop improvement. Poor people suffer from deficiencies in the essential amino acids like Lys and methionine. If crop plants contain less Lys, the nutritional value of such crops is also reduced by more than 50% and leads to imbalances in the amino acids. But breeding methods have led to the accumulation of Lys in vegetative tissues, which is in fact deleterious to the growth of plants (Ghislain et al., 1995). Therefore, pathways for Lys synthesis in plants have been identified and also the corresponding genes that encode them, keeping in view of their genetic transformation as an alternative approach. But such an effort requires identification of seed specific promoters, which help in the accumulation of more Lys in seeds/grains (Amir and Tabe, 2006). Most importantly, Lys acts as a precursor for the metabolic pathway implicated in plant stress response and also its development as revealed from the studies of Arruda et al. (2000) and Galili et al. (2001). Negrutiu et al. (1984) isolated for the first time, a Lys over-producing mutant from the protoplast cultures of *Nicotiana sylvestris*. This mutant was a result of altered expression of one of the biosynthetic pathway genes encoding a dihydrodipicolinate synthase (DHDPS). Reduced sensitivity to feedback inhibition in transgenic plants with overexpressed bacterial DHDPS (Perl et al., 1992; Shaul and Galili, 1992, 1993) helped to discover the Lys biosynthetic pathway and its modulation. Further, Karchi et al. (1995) dissected out that Lys biosynthesis and catabolism are coordinately regulated during the development of a seed in tobacco. Thus, for the first time the amino acid Lys has been correlated with plant development. Galili et al. (2001) later reviewed the catabolic events of Lys and implicated them not only to development but also to abiotic stress tolerance. A large body of information suggests that the regulatory networks associated with Lys biosynthesis and catabolism are intertwined largely with plant tissues and organ specificity and interactions between diverse metabolic fluxes.

Another important amino acid in the body of plants is serine (Ser), which helps to form the phospholipids necessary for signal transduction. L-Ser is not only a proteinogenic amino acid, but also it participates in catalytic functions of diverse enzymatic reactions in plants. Importantly, Ser takes an active part in the biosynthesis of many biomolecules such as amino acids, phospholipids, and sphingolipids that are obligatory for cell proliferation (Kalhan and Hanson, 2012). Ser gets phosphorylated by kinases and participates in signaling mechanisms (Chaneton et al., 2012). In plants, multiple biosynthetic pathways exist for Ser. The photorespiratory glycolate pathway appears as the major one, but non-photorespiratory phosphorylated pathway also exists.

This review discusses dehydration proteins known as dehydrins (DHNs), which are members of the LEA protein family group 2 or D-11 (Close, 1997). Since they do not have proper tertiary structures, they are described as “protein clouds” or “cooked spaghetti” (Uversky, 2013, 2016). DHNs are hydrophobic, with more than 50% of the residues being charged or polar in nature and 25% being either alanine or glycine. DHNs contain Lys- and Ser-rich residue and are associated with stress tolerance (Malik et al., 2017). Both Lys and Ser are essential, the former for humans, and the later for plants. Interestingly, both of them are important in plants as a link between metabolism and development. This review focuses on the climacteric links that have been established in recent times about Lys and Ser biosynthesis and catabolism, their association with plant growth and development, abiotic stress tolerance, and also Lys- and Ser-rich proteins, functional significance, and their remarkable ability to bestow stress tolerance in plants.

## Two Lys Biosynthetic Pathways Exist in Plants but Differ From That of Prokaryotes

**Figure 1:**
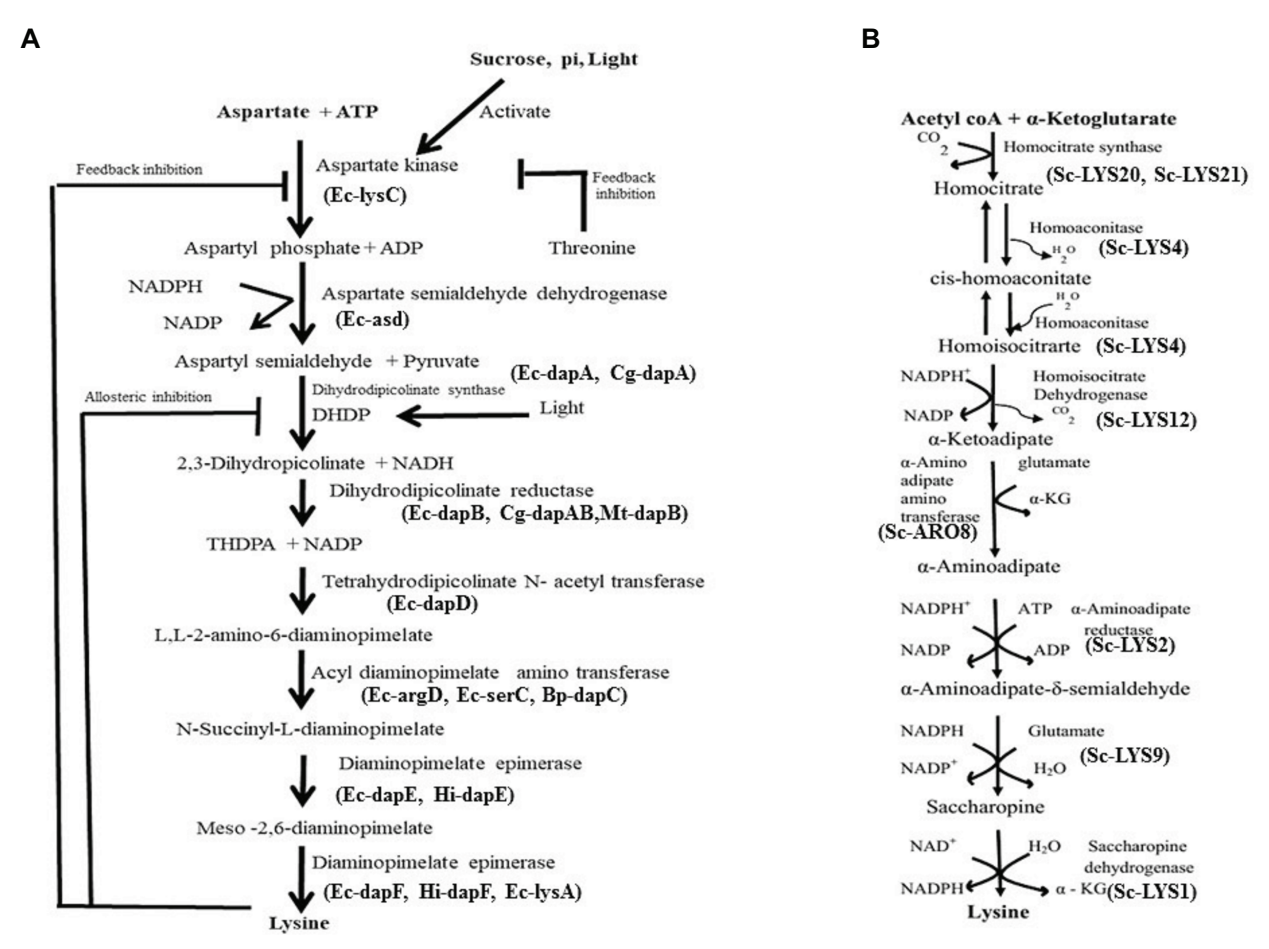
**(A)** Lysine biosynthesis pathway and its feedback regulation in plants. Ec, *Escherichia coli*; Sc, *S. cerevisiae*; Hi, *Haemophilus influenzae*. **(B)** Biosynthesis pathway of lysine *via* aminoadipate. The pathway operates mostly in fungi.

In nature, two pathways namely diaminopimelate (DAP) pathway (Figure 1A) and α-aminoadipate (AAA) have been identified for biosynthesis of Lys (Figure 1B). The first pathway belongs to the aspartate derived biosynthetic family, involved also in the synthesis of threonine, methionine, and isoleucine (Galili, 1995; Velasco et al., 2002; Hudson et al., 2005). The second pathway is part of the glutamate biosynthetic family, involving α-ketoglutarate and acetyl coenzyme A (as seen in *Thermus thermophilus*; Miyazaki et al., 2004; Xu et al., 2006). While the DAP pathway operates in prokaryotes and plants, the AAA pathway in yeast, protists, and higher fungi. In the DAP pathway, aspartate and pyruvate act as precursors for the biosynthesis of Lys *via* the intermediate DAP. This pathway adds carbon groups to aspartate to yield Lys (Figure 1A), but exhibits feedback regulation either by Lys or by threonine (Velasco et al., 2002; Hudson et al., 2005). Both pathways are not known to operate in any single organism/plant till date. In higher plants, Lys is synthesized in plastids and no evidence exists for its cytosolic synthesis (Hudson et al., 2006). Glutamate first gets converted to aspartate by aspartate aminotransferase enzyme (AAT). Aspartate kinase (AK), the first enzyme of the pathway catalyzes aspartate to 3-aspartic semialdehyde (3-ASA). While light and photosynthetic activity in the daytime positively affect 3-ASA biosynthesis especially in young leaves (Figure 1A), darkness stimulates the degradation of aspartate to asparagine. Isoenzymes of AK exist as monofunctional polypeptides, which have the Lys-sensitive kinase activity. Interestingly, the bifunctional enzymes AK/homoserine dehydrogenase (HSD) contain threonine-sensitive AK as well as HSD isozymes linked on a single polypeptide. Threonine partially feedback regulates HSD enzyme and, therefore, Lys production is affected (Galili, 2002; Figure 1A). Some of the factors affecting Lys biosynthesis in plants are shown in Table 1. Vauterin et al. (1999) have shown that activity of DHDPS is high in meristems, in the vasculature of leaves, roots, stems, carpels, styles, stigma, pollen, and young embryos. In line with this, they demonstrated that DHDPS promoter exhibits cell type-specific expressions, indicating its multiple functions in diverse tissues. Synthesis of Lys by transcriptional activation in a tissue specific manner clearly implicates regulation of the enzyme at the RNA level. The enzyme *L,L*-diaminopimelate aminotransferase (_LL_-DAP-AT) is a novel variant that catalyzes tetrahydrodipicolinate to *L,L*-DAP and then helps in the synthesis of Lys in lower plants (*Physcomitrella patens*) as well as higher plants like *A. thaliana*, soybean, and spinach (Hudson et al., 2006). This indicates that the enzyme is highly conserved among diverse plant species. Further, in *A. thaliana*, this enzyme catalyzes a reversible reaction. DHDPS and dihydrodipicolinate reductase (DHDPR) enzymes catalyze the two vital steps in Lys biosynthesis (Figure 1A). It is arguable, however, if it has a tetrameric or dimeric arrangement since it is not yet distinctly known. Tetrahydrodipicolinate (THDPA) is converted to *meso*-2,6-diaminopimelate (*m*-DAP) catalyzed by different enzymes. Hudson et al. (2006) reported a novel and specific _LL_-DAP-AT that directly converts THDPA to _LL_-DAP in *A. thaliana* (regarded as DAP-AT pathway). Interestingly, _LL_-DAP-AT operates efficiently in the forward/biosynthetic direction in plants (Hudson et al., 2006). Diaminopimelate epimerase is the last enzyme in the pathway, which converts *m*-DAP to Lys (Figure 1A).

**Table 1:** Factors affecting the biosynthesis of Lys and Ser in plants.

Lysine	Serine	References
Two pathways exist in plants for the biosynthesis of Lys.	Three (glycolate linked to photorespiration and two non-photorespiratory) pathways exist for Ser biosynthesis.	Galili (2002), Zhu and Galili (2004), Ros et al. (2014), Dellero et al. (2016), Igamberdiev and Kleczkowski (2018).
Synthesized in plastids.	Synthesized in chloroplasts and mitochondrial matrix.	Ho and Saito (2001).
Feedback regulation by Lys controls its own synthesis. Further, threonine also affects Lys synthesis. Thus, two amino acids control its biosynthesis. Lys also inhibits DHDPS and arrests thereby its own biosynthesis.	Feedback regulation by Ser controls its biosynthesis, but activated by another amino acid L-homocysteine unlike Lys, suggesting an allosteric mechanism.	Galili (2002), Velasco et al. (2002), Ros et al. (2014), Zhu and Galili (2004), Okamura and Hirai (2017).
Lys production is concurrently regulated by both synthesis and degradation in reproductive and vegetative tissues.	Ser production in photosynthetic tissues is mostly regulated by high atmospheric CO_2_ concentration.	Zhu and Galili (2004), Ros et al. (2014).
Light and photosynthetic activities affect the first enzyme AK in the pathway positively, and darkness stimulates degradation of aspartate to asparagine. Lys inhibits its synthesis by inhibiting the activity of AK. AK is also sensitive to threonine. Lys allosterically inhibits DHDPS, thereby arresting its own synthesis.	Interaction of Fd-GOGAT has been found essential for photorespiratory SHMT activity. Therefore, a complex regulation occurs in this high flux pathway, and Ser biosynthesis.	Galili (2002), Hudson et al. (2006).
Two genes in the pathway are light-regulated. AK is also modulated by sucrose and inorganic phosphate (Pi), thus affect Lys biosynthesis.	Expression of only *PGDH1* is modulated by high CO2, but not by *PGDH2*. Both *PGDH1* and *PGDH3* genes are regulated by light-dark regimes in photosynthetic tissues. In non-photosynthetic tissues, PGDH genes are expressed under light-dark regimes. Thus, PPSP pathway appears critical for both types tissues and affects thereby Ser biosynthesis.	Zhu-Shimoni and Galili (1998), Vauterin et al. (1999), Galili (2002). Benstein et al. (2013), Toujani et al. (2013).

## Transgenic Plants Accumulate High Lys Content

Transgenic lines were generated by over expressing bacterial Lys biosynthetic pathway genes in several plants. While seeds were wrinkled in high-Lys producing transgenic *Glycine max* (Falco et al., 1995), seed germination retarded in *A. thaliana* (Zhu and Galili, 2003, 2004), and the endosperm hardened in the *QPM* mutant of maize (Gibbon et al., 2003). Long et al. (2013), and Yang et al. (2016) generated transgenic rice by overexpressing a combination of bacterial genes that encode AK and DHDPS and also by inhibiting Lys ketoglutarate (α-KG) reductase/saccharopine dehydrogenase (LKR/SDH). These transgenic lines of rice displayed 60-fold increase in Lys and thus biofortified, but endosperm color was dark-brown. Surprisingly, the seed germination and subsequent performance of these plants when evaluated in the field conditions did not show any change in their nutrition. Overexpression of tryptophan decarboxylase 3, an enzyme involved in serotonin (sometimes called the happy chemical, seen in human brain mostly) biosynthesis proved that tryptophan (a remote precursor of serotonin) and serotonin are responsible for dark-brown phenotype of endosperm in high-Lys producing lines (Kanjanaphachoat et al., 2012; Yang et al., 2016, 2018). They also proposed that 2-aminoadipate (an intermediate in Lys catabolism) might play a role between jasmonate signaling, elevated serotonin levels, and the endosperm color. Using a seed-specific promoter, Zhu and Galili (2003, 2004) overexpressed a bacterial *DHDPS* gene in a knockout *A. thaliana* mutant lacking the bifunctional enzyme α-aminoadipic semialdehyde synthase (AASS) that contains both LKR/SDH activities (in bacteria and fungi, the two enzymes are encoded by separate genes; Zhu et al., 2002). Transgenic *A. thaliana* seeds displayed 64-fold increase in free Lys levels. Houmard et al. (2007) achieved significant increase in the amount of Lys in transgenic maize kernels by RNA interference technique through endosperm specific suppression of *LKR/SDH* gene. Possibilities exist therefore for attaining high Lys containing crop plants as nutrient/food supplements to both animals and humans using its degradative pathway genes. In the light of the above facts, a deeper understanding of the metabolism and its networks are needed before we create Lys/nutrient-rich crop plants acceptable to the consumers.

## Lys Catabolism Produces Glutamate and Stress-Related Metabolites, Which Help to Overcome Abiotic Stress

**Figure 2:**
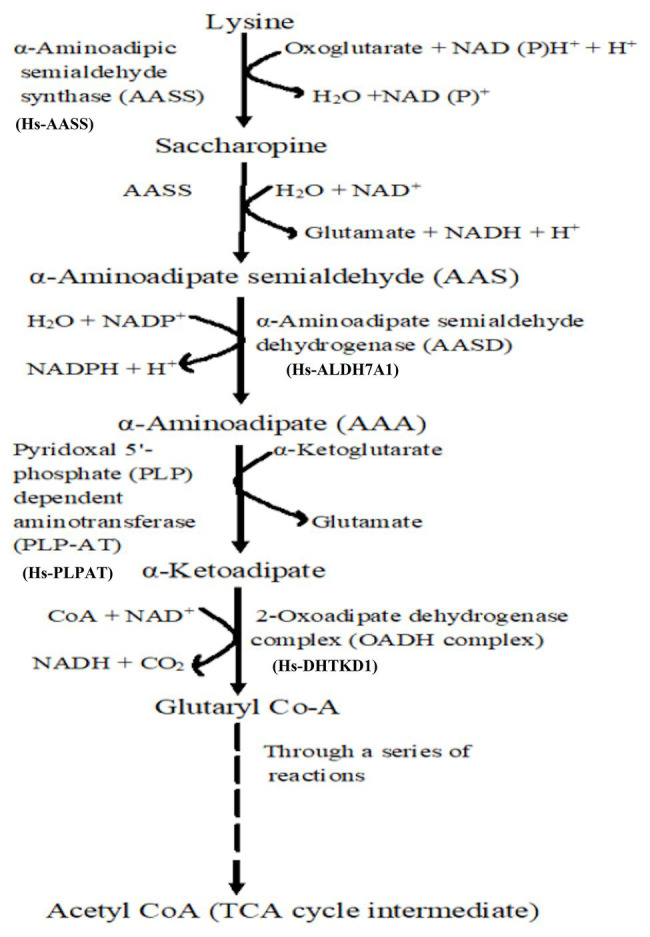
Lysine catabolism *via* saccharopine is the prominent pathway. Broken line indicates that through several reactions, glutaryl Co-A is converted to TCA cycle intermediate acetyl coenzyme A for energy generation.

If amino acids (cysteine or Lys for example) are accumulated in high concentrations, they may be toxic to the plants (Zhu and Galili, 2004), hence, they must be degraded. The saccharopine pathway (Figure 2) is the primary pathway for Lys degradation, which occurs in the liver (in animals) specifically within mitochondria (Galili et al., 2001). This is the reverse of the AAA pathway (Figure 1B), and in plants and animals, the first two steps in the catabolism are catalyzed by LKR/SDH activities. Lys combines with α-KG to form saccharopine by the enzyme LKR (Figure 2), which ultimately generates glutamate and other stress-related metabolites. Also, Lys has been found vital during seed germination, where it serves as a source of carbon and energy by feeding the intermediates (like α-KG) of TCA cycle to the growing seedlings (Afendi et al., 2012). Thus, the mechanistic insights into Lys degradation and its intermediates serving as the precursors for generation of energy (NADH) *via* the electron transport chain have been furnished (Araujo et al., 2010).

Lys serves as an alternative respiratory substrate under a variety of stresses, including drought, and senescence under extended darkness (Urano et al., 2009; Joshi et al., 2010). Flexibility/remobilization of molecules are necessary since it helps the plants to maintain metabolic homeostasis and supply energy, the primary necessity to survive. Consistent with this, under salinity and drought stress conditions, contents of Lys and other amino acids increased in wheat, potato, and safflower, which displayed tolerance to stress (Saeedipour and Moradi, 2012; Muttucumaru et al., 2015; Zafari and Ebadi, 2016) and high expression of *LKR/SDH* and aminoadipic semialdehyde dehydrogenase (*AASADH*) genes involved in saccharopine pathway (Brocker et al., 2010; Less et al., 2011). Further, ectopic expression of *AASADH* resulted in the generation of stress-tolerant plants (Rodrigues et al., 2006). In radish, drought stress was relieved with Lys application in chelation with Zn as seed priming (Noman et al., 2018). Supporting such studies, Wang et al. (2013) observed higher Lys and protein contents in transgenic maize lines in comparison with wild type plants. Such transgenics exhibited not only substantial growth but also salt stress tolerance. Upregulation of saccharopine pathway genes directs Lys to degrade to α-aminoadipate semialdehyde (AAS) and glutamate, which in turn regulates homologs of animal glutamate receptors involved in plant development (Galili et al., 2001). Arruda et al. (2000) pointed out that the saccharopine pathway described above might be accountable for the biosynthesis of several regulatory molecules implicated in root growth and senescence under both biotic and abiotic stresses. Further, Rizwan et al. (2017) demonstrated the alleviation of metal stress in wheat upon foliar spray of Lys. It has been found that variability exists among different populations of *A. halleri* for Cd accumulation (Stein et al., 2017; Corso et al., 2018). Such an intraspecific variability has prompted Serre et al. (2020) to investigate the molecular mechanisms associated with metal tolerance, especially the Lys-methylated proteins in plants. The effect of Cd on Lys-methylated proteins and KMTs were studied in *A. thaliana*, *A. lyrata*, and *A. halleri*, which have differential tolerance to Cd. Gene expression, protein mass spectrometric, and immunoblotting techniques revealed significant expressions in Lys-methylated proteins and regulation of genes coding KMTs by Cd (Serre et al., 2020). Interruption of KMT gene in *A. thaliana* displayed a significant increase in Cd tolerance in comparison with wild type plants. Further, a knock-out mutant of the calmodulin Lys methyltransferase gene exhibited enhanced Cd tolerance. These results suggest that the regulation of nonhistone proteins by Lys methylation play pivotal roles in *A. thaliana* plants to Cd stress. The results are novel and unexpected, hinting us to explore more about lysine-methylated proteins and KMTs in future to generate plants for phytoremediation. But in the developing maize endosperm, saccharopine pathway is induced by exogenous supply of lysine and repressed by salt stress. In young maize coleoptiles, *LKR/SDH* and *AASADH* were induced at the transcript level by abiotic stress. Only the AASADH protein accumulates in the stressed tissues, but not the LKR/SDH. These results indicate that in the developing seeds, the saccharopine pathway is used for pipecolic acid synthesis, although proline plays a role in abiotic stress response. Thus, saccharopine pathways in maize seed development and stress response differ from dicots (Kiyota et al., 2015). Batista-Silva et al. (2019) demonstrated rapid detoxification of Lys in the leaves of *A. thaliana* during initial stages of stress recovery. Their results indicate that amino acid-derived secondary metabolites increase when the stress is being relieved and further showed an increase in the levels of amino acids that act as precursors for secondary plant product biosynthesis.

## Multiple Pathways Co-Exist for Ser Biosynthesis in Plants

**Figure 3:**
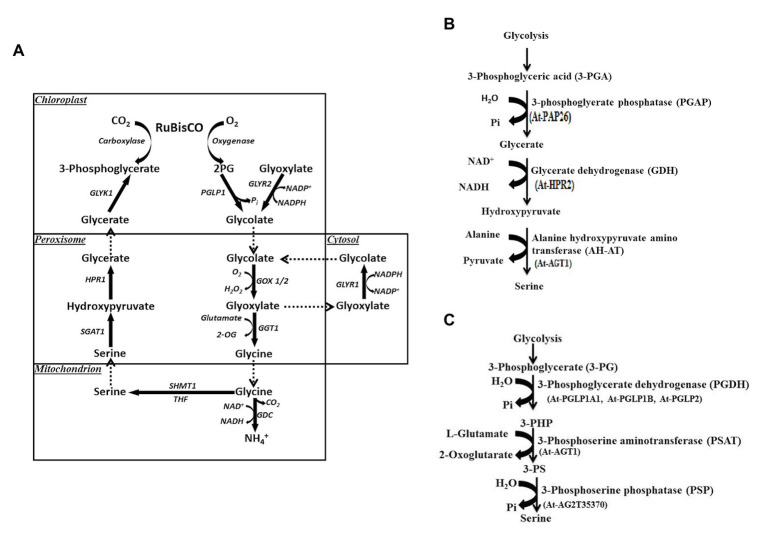
**(A)** Biosynthesis of serine *via* glycolate pathway during photorespiration. 2PG, 2-phosphoglycolate; PGLP1, 2PG phosphatase 1; GLYR1/2, glyoxylate reductase 1/2, glutamate glyoxylate aminotransferase 1; GDC, glycine decarboxylase complex; THF, tetrahydrofolate; SHMT1, serine hydroxymethyl transferase 1; SGAT1, serine glyoxylate aminotransferase 1; HPR, hydroxypyruvate reductase 1; GLYK1, glycerate kinase 1. **(B)** Serine biosynthesis *via* glycerate pathway. At, *Arabidopsis thaliana*. **(C)** Phosphorylated pathway of L-serine biosynthesis (PPSB). 3-PG, 3-phosphoglycerate; 3-PHP, 3-phosphohydroxypyruvate; 3-PS, 3-phosphoserine; and At, *Arabidopsis thaliana*.

Ser participates in the biosynthetic pathways of several key biomolecules essential for synthesis of other nitrogen bases (purines, pyrimidines, and thymidine), amino acids (cysteine, glycine, and tryptophan), phospholipids, sphingolipids, and cell proliferation (Ros et al., 2014; Mattaini et al., 2016). Cysteine in turn is needed for the biosynthesis of both methionine and homocysteine. Ser biosynthesis in plants proceeds *via* different pathways: glycolate pathway, which is associated with photorespiration and two non-photorespiratory pathways and the phosphorylated and the glycerate pathways. Existence or coexistence of three distinct pathways in plants for Ser biosynthesis reveals the complex nature of its regulatory processes. Out of the three, glycolate pathway (Figure 3A) has been considered as the prime pathway (Tolbert, 1980; Douce et al., 2001). Photorespiration has been considered as the main source of Ser in plants since this pathway is associated with it; therefore, it has been assumed that, at least, in photosynthetic tissues the quantitative contribution of this pathway to Ser pool would be higher. For this reason, the non-photorespiratory pathways have been considered of minor importance to date. Ser is synthesized through glycolate, glycerate, and PPSB pathways, which are described below.

## Biosynthesis of Ser in Plants

Through the glycolate pathway, L-Ser is synthesized during plant photorespiration within the mitochondrial matrix. In this pathway, one molecule of glycine is decarboxylated and deaminated by a fascinating complex enzyme called glycine decarboxylase (GDC). Both carbon dioxide (CO_2_) and ammonia (NH_3_) are formed with the release of NADH from NAD^+^ (Douce et al., 2001). In a complex reaction, the methylene carbon of glycine molecule is transferred to tetrahydrofolate (THF) to form methylene tetrahydrofolate (5,10-CH_2_-THF). Methylene THF now combines with the second molecule of glycine to form L-Ser catalyzed by a Ser hydroxymethyltransferase (SHMT) enzyme (Figure 3A). This is a pyridoxal 5'-phosphate-dependent enzyme, which catalyzes the reversible reaction of Ser to glycine in either a tetrahydrofolate-dependent or tetrahydrofolate-independent manner. One-carbon units that result from the activity of SHMT, have been found vital in proliferating cells (Wu et al., 2017). The glycolate pathway has also biological significance like the other two. For instance, *Arabidopsis* SHMT mutant (*shm1-1*) was isolated by Somerville and Ogren (1981), which shows a strong photorespiratory phenotype that is rescued under high CO_2_ (Voll et al., 2006). One of the *SHMT1*-deficient mutants defective in *GLU1* gene has been reported in *A. thaliana* (Jamai et al., 2009), which encodes ferredoxin-dependent glutamate synthase located in chloroplasts (Fd-GOGAT). This enzyme is associated with the photorespiratory reassimilation of NH_3_ and primary nitrogen assimilation. An important observation made by Jamai et al. (2009) shows that Fd-GOGAT is targeted to the mitochondria as well as chloroplasts. Interaction of Fd-GOGAT has been found essential for photorespiratory SHMT activity (Table 1). Thus, a complex regulation occurs in this high flux pathway. Both SHM1 and SHM2 are targeted to the mitochondria and operate redundantly in one carbon metabolism of non-photorespiring especially during lignification of vascular cells. Interestingly, double mutant lacking both mitochondrial SHMTs shows a lethal phenotype even under non-photorespiratory conditions, which point out the non-redundant role(s) of both isoforms and the importance of C1 metabolism for plant development (Engel et al., 2011). Five more SHM isoforms were predicted and targeted to different subcellular compartments like cytosol, plastids, and nucleus. Functional information is available for the plastidic isoform SHM3 (Zhang et al., 2010), which is believed to be involved in general one-carbon metabolism along with cytosolic isoforms SHM4 and SHM5 (Engel et al., 2011).

Ser is also synthesized in leaves through glycerate pathway. It is well-known that 3-phosphoglycerate (3-PG) is synthesized during glycolytic pathway (Figure 3B). L-Ser is formed from 3-PG by a dephosphorylation reaction (Kleczkowski and Givan, 1988) and includes part of the reverse sequence of the photorespiratory cycle. 3-PG forms glycerate and the reaction is catalyzed by 3-phosphoglycerate phosphatase (PGAP). Glycerate is then converted to hydroxypyruvate by glycerate dehydrogenase (GDH) enzyme (Figure 3B). In the last reaction, alanine-hydroxypyruvate aminotransferase (AH-AT) enzyme and glycine hydroxypyruvate aminotransferase (GH-AT) convert hydroxypyruvate to L-Ser. While one reaction takes place in the cytosol (PGAP), the remaining three (GDH, AH-AT, and GH-AT) take place in peroxisomes through enzymatic pathways (Kleczkowski and Givan, 1988; Greenler et al., 1989). To date, several genes involved in the glycerate pathway have been identified. However, no specific genes have been characterized and/or cloned to account specifically for glycerate pathway, so its functional significance remains little unclear (Ros et al., 2013; Igamberdiev and Kleczkowski, 2018).

One of the Ser biosynthetic pathways, PPSB, is conserved across bacteria, plants, and animals (Fell and Snell, 1988; Ho and Saito, 2001) and operates in plastids in plants. 3-PG generated *via* plastidial glycolysis and Calvin cycles acts as a precursor for the synthesis of Ser (Figure 3C). In the three sequential reactions, 3-PG is first converted to 3-phosphohydroxypyruvate (3-PHP) by the enzyme D-3-phosphoglycerate dehydrogenase 1 (PGDH1), EC 1.1.1.95; Slaughter and Davies, 1968). In this conversion, the cofactor NAD^+^ is reduced to NADH. 3-PHP is then converted to 3-phosphoserine (3-PS) by a transamination reaction catalyzed by 3-phosphoserine aminotransferase (PSAT, EC2.6.1.52; Hanford and Davies, 1958). Wulfert and Krueger (2018) for the first time provided the genetic evidences for the *PSAT* genes. *PSAT1*-silenced lines displayed a strong inhibition of shoot and root growth and hypersensitivity to the inhibition of the photorespiratory Ser biosynthesis at higher CO_2_ levels (Table 1). Such lines also showed accumulation of certain amino acids, due to increased assimilation of NH_3_. Knockdown of *PSAT1* alters the amino acid metabolism in plants (Wulfert and Krueger, 2018). PSAT catalyzes the transfer of the amino group of glutamate to 3-hydroxypyruvate, resulting in 2-oxoglutarate and 3-phosphoserine. It is interesting to note that *PSAT1* is essential for light and sugar-dependent growth promotion in *A. thaliana* (Wulfert and Krueger, 2018). During this biosynthetic pathway, glutamate is converted to 2-oxoglutarate, an intermediate in the TCA cycle that generates energy. 3-PS is finally catalyzed by 3-phosphoserine phosphatase (PSP, EC 3.1.3.3) to form Ser (Figure 3C). The enzymatic and genetic evidence for these enzymes were achieved by Benstein et al. (2013), Cascales-Miñana et al. (2013), and Toujani et al. (2013). While three genes for PGDHs were described, two genes encoding for PSAT noticed in the TAIR,^1^ cloned and observed to have activity *in vitro* (Ho et al., 1998, 1999a,b; Ho and Saito, 2001). Coexistence of multiple genes for PPSB and the presence of corresponding enzymes in different tissues indicate that they may perform diverse functions. This fact needs to be further investigated, but there is evidence supporting it (Ho and Saito, 2001; Waditee et al., 2007; Benstein et al., 2013). The genes associated with PPSB are expressed in several tissues (Ros et al., 2014) based on which it is argued that this pathway acts as a link between metabolism and development. It has been demonstrated that *PGDH1* expression has been modulated by high CO_2_ levels (Benstein et al., 2013). They demonstrate that the content of Ser is reduced in *PGDH1*-silenced plants exposed to high CO_2_. They are of the opinion that expression of PGDH_2_ is not modulated by high CO_2_. Furthermore, *PGDH1* and *PGDH3* genes have been found regulated in *A. thaliana* by light-dark regimes in photosynthetic tissues (Toujani et al., 2013; Table 1). On the other hand, in non-photosynthetic tissues, *PGDH* family of genes are equally expressed under light-dark regimes indicating that PPSB pathway (Figure 3C) may be critical for both photosynthetic and non-photosynthetic tissues (Table 1). Unlike *PGDH* family of genes, where their expression pattern is tightly regulated at organ level, *PSP1* gene is expressed in all organs (Cascales-Miñana et al., 2013; Toujani et al., 2013). This could be due to the presence of a unique *PSP1* gene. Thus, several pieces of evidence point out that the PPSB pathway is highly relevant in photosynthetic tissues during dark conditions, when photorespiration does not operate.

## Ser Metabolism is Essential for Plant Development and Abiotic Stress Tolerance

Though biological significance of PPSB has been demonstrated and found essential for the development of male gametophyte, pollen, embryo, and postembryonic root growth (Cascales-Miñana et al., 2013; Toujani et al., 2013; Ros et al., 2014, and the references therein), its metabolic implications have not been completely understood. In PPSB-deficient mutants, adenosine 5'-phosphosulfate reductase genes, sulfate transporters were upregulated (Anoman et al., 2019) with enhanced flux of ^35^S into thiol biosynthesis, mostly in root tissues, and also their transport and allocation. Thus, deficiency of PPSB activity perturbs sulfur homeostasis between photosynthetic and non-photosynthetic tissues (Anoman et al., 2019). Besides supplying carbon to the one-carbon pool (necessary for the biosynthesis of thymidylate and methionine), Ser is implicated in signaling mechanisms (Ser is phosphorylated by kinases; Antonov et al., 2014; Ros et al., 2014; Mattaini et al., 2016). Double mutants of *gapcp1gapcp2* arrested not only primary root growth, but also showed defects in pollen development. Supplementation of L-Ser to the *gapcp1gapcp2* mutant roots resulted in the recovery of root growth (Muñoz-Bertomeu et al., 2009). Further, it has been noticed that embryo and male gametophyte become lethal in mutant plants lacking Ser palmitoyltransferase, an enzyme associated with sphingolipid biosynthesis (Chen et al., 2006; Dietrich et al., 2008). Associated with the above, in *psp1* and *pgdh1* homozygous mutant, development of embryo in *Arabidopsis* is totally arrested inferring the importance of Ser in all the above cases (Benstein et al., 2013; Cascales-Miñana et al., 2013; Toujani et al., 2013). Similarly, in *PSP1*- and *PGDH1*-deficient *Arabidopsis* mutants, root development was inhibited, indicating poor auxin biosynthesis in such mutants. In plants, tryptophan (produced by condensation of Ser and indole) acts as a precursor for auxin biosynthesis (Tzin and Galili, 2010; Dunn, 2012), so lack of Ser in the cells leads to root developmental defects. Benstein et al. (2013) further noticed inhibition of leaf initiation when plants containing reduced levels of PGDH activity were grown under elevated levels of CO_2_. Ser is also implicated in folate metabolism. Mutants deficient in tetrahydrofolate metabolism show defects both in root and embryo development. It is however opined that Ser is not solely responsible for root development, and multiple factors/processes might be involved along with Ser. Interestingly, D-Ser (a derivative of L-Ser) has been found to act as a signaling molecule between male gametophyte and pistil communications (Michard et al., 2011). Thus, these observations infer that Ser metabolic pathways are critical for plant ontogeny. Alterations in *PPSB* gene expressions perturb not only the TCA cycle but also amino acid biosynthesis (Cascales-Miñana et al., 2013; Toujani et al., 2013).

*SHMT1* functions in the photorespiratory pathway and play a critical role in controlling cell damage provoked by high light (photooxidative stress) and salt stresses and in restricting pathogen-induced cell death (Moreno et al., 2005). Further, SHMT1, located in mitochondria, converts glycine to Ser, and Liu et al. (2019) reported complementation of *A. thaliana shm1-1* (photorespiratory growth phenotype) by SHMT1 wild type plants. They found that reduced SHMT activity led to a decreased stomatal closure in response to ABA and salt stress. Transgenic lines, which showed reduced *SHMT* activity, were not only more sensitive to salt stress but also to drought stress recovery. In line with this, Batool et al. (2018) propose that cysteine is the limiting factor for ABA biosynthesis in the early stages of drought conditions in guard cells and potentially in other cell types of the leaf. Thus, its explicit glycolate pathway has a role to play in plant abiotic stress. Glycerate pathway has been linked to γ-aminobutyric acid (GABA) shunt, which affects plant growth as well as development throughout life cycle and accumulates rapidly and contributes in response to biotic and abiotic stresses (Ramos-Ruiz et al., 2019). Ser formed in glycerate and PPSB pathways acts as a precursor of glycine, formate, and glycolate, which accumulate under stress conditions (Igamberdiev and Kleczkowski, 2018). A hypothesis has been presented by Igamberdiev and Kleczkowski (2018) on the regulation of redox balance in stressed plant cells *via* participation of the reactions connected with glycerate and PPSB pathways. Thus, a link between carbon and nitrogen metabolism exists, which is regarded as vital in maintaining cellular redox and energy levels under stress conditions.

Accumulation of Ser was noticed in plants exposed to low and high temperatures, flooding, and high salt stress levels (Stewart and Larher, 1980; Kaplan et al., 2004; Bocian et al., 2015; Li et al., 2017) as well as combined stresses of drought and heat (Hossain et al., 2017). Overexpression of *PGDH* gene isolated from *Aphanothece halophytica* (a cyanobacterial species) lead to elevated salt and cold stress tolerance in *A. thaliana* (Waditee et al., 2007), indicating the involvement of PGDH enzyme in abiotic stresses. In line with this, studies conducted by Rosa-Téllez et al. (2020) have demonstrated that the response observed to salt stress depends on the isoform studied. Thus, while *PGDH1* activity could be relevant for plant tolerance to salinity, the function of *PGDH3* seems detrimental under such environmental conditions. Lines overexpressing *PGDH1* (*OexPGDH1*) accumulate less proline and raffinose (stress markers) in roots under salt stress than lines overexpressing *PGDH3* (*OexPGDH3*). Moreover, the levels of oxidized glutathione (GSSG; derived from Cys) increased under salt stress in *OexPGDH3* as compared to both *OexPGDH1* and wild type plants. Ser is utilized in many biosynthetic pathways and thereby contributes to nucleotide synthesis, methylation reactions, and production of the reducing power. Both glutathione and NADPH help in antioxidant defense in plants (Yang and Vousden, 2016). Glutathione acts as a precursor of phytochelatins and helps in chelating toxic metals, and needed for the detoxification of methylglyoxal, a cytotoxic and an emerging signaling molecule in plant abiotic stress responses and tolerance. Further, glutathione has been found to impact translation and subsequent changes. Interestingly, PPSB could affect the ABA signal transduction and thus trigger the downstream genes under cold and salt stress conditions. In support of this statement, double mutants like *gapcp1gapcp2*, which have an impaired phosphorylation pathway are insensitive to ABA (Muñoz-Bertomeu et al., 2011a,b), suggesting that Ser pathway somehow triggers ABA signals for environmental stress tolerance. Ser acts as a precursor for the synthesis of glycine betaine, an important osmotic agent, and a scavenger of ROS. Some species like rice and *Arabidopsis* do not produce glycine betaine, but in such a case, glutathione (a derivative of cysteine) scavenges ROS generated during abiotic stress (Rosa-Téllez et al., 2020). The relevance and importance of catabolism of amino acids as an alternative respiratory substrate has been demonstrated during drought or short light periods (Araujo et al., 2010; Engqvist et al., 2011; Krüßel et al., 2014). It appears therefore that a tight relationship exists between Lys and Ser metabolism during abiotic stress recovery.

## DHNs Contain Lysine-Rich Residues Involved in Abiotic Stress Tolerance

Late embryogenesis abundant proteins (LEAs) are hydrophilic and thermostable in nature. This property helps them to interact not only with biomolecules, but also metal ions (Liu et al., 2013; Cuevas-Velazquez et al., 2017). DHNs are group II LEA proteins and are widely distributed in bryophytes, gymnosperms, and angiosperms (Yu et al., 2018), accumulate during embryogenesis and induced in vegetative tissues following exposure to diverse environmental stresses (Battaglia et al., 2008). They are low molecular weight (ranging 9–200 kD), modular, intrinsically disordered proteins (IDPs), and lack well-defined three-dimensional structures (Riley et al., 2019). But SbDHN5 (from *Sorghum bicolor*) has been discovered as an ordered protein with phosphorylation sites (Nagaraju et al., 2018). Hence, it would be of interest to find out the overexpression of *SbDHN5* in crop plants and their resistance to abiotic stresses. Proximate *in vitro* evidences exist that DHNs protect plants in myriad ways, such as buffering of ion sequestration (Alsheikh et al., 2003), hydrate water (Bokor et al., 2005), chaperone activity (Abedini et al., 2017), membrane binding and stabilization (Xing et al., 2011), enzyme cryoprotection (Hughes and Graether, 2011; Drira et al., 2013), and in scavenging ROS (Hara et al., 2013). DHNs contain Lys-rich K-segment, which is prevalent in all DHNs with EKKGIMDKIKEKLPG (15 amino acids) and located near the C-terminus (Close, 1996; Malik et al., 2017). K-segments participate in forming class A2 amphipathic α-helix that protect both enzymes and membranes (Baker et al., 1988). More than one K-segment is also noticed in few DHN proteins, but with several conserved residues (Koag et al., 2009; Graether and Boddington, 2014). DHNs have four different types of conserved sequence motifs namely K-, S-, Y-, ([V/T]D] [E/Q]YGNP), and ф segments. Quite intriguing is that no position in the K-segment is conserved totally (Graether and Boddington, 2014). Without any exception, all DHNs contain a minimum of one copy of Lys-rich K-segment located near the C-terminal. In some DHNs, more than one K-segment, but with distinct amino acid sequences may be present. The conserved residues among the K-segment include Lys-Ile-Lys-Glu in the core, Lys-Leu-Pro-Gly in the C-terminal, and Glu-Lys-Lys in the N-terminal regions (Graether and Boddington, 2014). However, the amino acid residues in K-segment among lower and higher plants may differ. In gymnosperms, K-segment shares a variable sequence like (Q/E)K(P/A)G(M/L)LDKIK(A/Q)(K/M)(I/L)PG, while in higher plants, it has EKKGIMDKIKEKLPG (Close, 1996; Jarvis et al., 1996). While K-segment is associated with plant development besides stress tolerance, the conserved sequence of Y is homologous to that of chaperone molecules (Martin et al., 1993). On the other hand, S-segment contains Ser cascade sequence SSSSSSSD, and this segment is observed mostly as a single copy in DHNs. S-segment is a phosphorylatable patch of 4–10 Ser residues and can transfer DHNs from cytoplasm to the nucleus (Goday et al., 1994). Stretches of sequence with a variable length are usually noticed and S-segment also participates in plant development alongside abiotic stress tolerance. It is also of interest to note that in Y_n_SK_n_-type DHNs, the K- and S-segments are linked by a fixed motif GXGGRRKK (where X can be any amino acid), indicating a functional linkage between K- and S-segments. While the motif GXGG is highly pliable and interacts with negatively charged phosphoserines with K-segment, motif RRKK appears to be a nuclear localization signal (Jensen et al., 1998; Malik et al., 2017). In between the conserved motifs, there are ф segments with small, polar, and charged amino acids. Ф-segments are stretches of sequences with variable lengths of amino acids. Based on the sequence and the number of K, S, and Y segments, DHNs have been further classified in five subfamilies/subgroups such as K_n_S, Y_n_SK_n_, K_n_, Y_n_K_n_, and SK_n_ (Mundy and Chua, 1988). While Y_n_SK_n_-type DHNs are expressed mostly under desiccation and salt stresses, K_n_, SK_n_, and K_n_S are upregulated by cold, desiccation, and salt stresses (Graether and Boddington, 2014). The presence of S (Ser) motif preceding the K (Lys) motif (Y_n_SK_n_, SK_n_, and S_n_KS) and also frequent occurrence of one of such subclasses Y_n_SK_n_ in monocots has been noticed by Abedini et al. (2017). Interestingly, out of 13 HvDHNs, 10 Y_n_SK_n_ members were noticed in a drought-tolerant barley (Kosová et al., 2011), and three in *Sorghum bicolor* out of six detected (Nagaraju et al., 2018). DHNs have been observed in vegetative tissues grown under control conditions, inferring that they play a key role in plant growth. The Y2K4-type DHN MtCAS31 (from *Medicago truncatula*) by interacting with AtICE1 (induces CBF expression 1) has been shown to associate with stomatal development, increasing the drought tolerance by decreasing the stomatal density of transgenic *A. thaliana* (Xie et al., 2012). DHN5 when overexpressed in *A. thaliana* showed different responses to biotic (as an antibacterial and antifungal factor) and abiotic stresses (Drira et al., 2015, 2016) besides protecting lactate dehydrogenase, β-glucosidase, and glucose oxidase from cold and heat stresses (Brini et al., 2010; Drira et al., 2013).

The DHNs are localized mostly in the cytoplasm, nucleus, plasma membrane, and mitochondria (Hara et al., 2013). It is interesting to note that many S-segment-containing DHNs are localized to the nucleus inferring that the S-segment moves to nucleus besides K_n_ and Y_n_K_n_ types (Wisniewski et al., 1999; Lin et al., 2012). Graether and Boddington (2014) discovered that several of K_n_, SK_n_, K_n_S, Y_n_SK_n_, and Y_n_K_n_ DHNs were upregulated during low temperature (cold), desiccation, and salt stresses. The His-flanking K-segments (the major functional component) have been found to bind to membranes and play a major role during stress response (Eriksson et al., 2011). Further, a correlation exists between the number of K-segments and abiotic stress tolerance. In line with this statement, K-segment of wheat DHN WZY2 has been found to protect plants from temperature stress (Yang et al., 2015). In this regard, it is interesting to note that the derivative containing two K-segments (WZY2) displays remarkable cold and heat stress tolerance than the truncated derivative without K-segments. This implies that K-segment is the major functional component of WZY2 (Yang et al., 2015). Interestingly, among the spliced DHN1a_s (YSK2) and unspliced DHN1a_u (YS), only the spliced variant exhibited resistance to cold and drought stresses and to *Botrytis cinerea* (Rosales et al., 2014).

## Ser-Rich Proteins are Implicated in Abiotic Stress as Well as in Plant Development

Lazar et al. (1995) first identified serine/arginine-rich (SR) proteins using monoclonal antibodies raised against a Ser phospho epitope in the arginine/serine-rich (RS) domain. SR proteins now appear as a highly conserved family of RNA-binding protein members in eukaryotes and regarded as crucial alternative splicing (AS) regulators of pre-mRNAs (Palusa et al., 2010), thereby increasing the transcript complexity. SR proteins range in size from 21 to 41 kDa in *A. thaliana*, and have two RNA recognition motifs at the N- and C-terminal RS domains, rich in SR dipeptides. AS has been recognized as a means of plant adaptation to a changing environment and is controlled in a tissue- and development-specific manner especially under abiotic stress conditions. These proteins play crucial roles in maintaining genome stability (Xiao et al., 2007), promoting transcriptional elongation (Lin et al., 2008), and cell cycle progression (Zhong et al., 2009). Many environmental stresses modulate AS patterns of SR proteins, phosphorylation status, and subcellular distribution in plants. In the promoter regions, Chen et al. (2019) found 92 development-, stress-, and hormone-related *cis*-elements. This implies that SR proteins play an important role during plant development and in response to environmental stresses. SR proteins were predicted to interact with other SR and non-SR proteins, inferring their association in other functions. Developmental defects were noticed with the overexpression of *Arabidopsis* SRp30, RSZ33, or mutations in SC35 and SCL genes (Lopato et al., 1999; Maria et al., 2003; Yan et al., 2017), implying the involvement of SR proteins in plant development. The number of SR proteins may vary in different taxa (16 in *Physcomitrella patens*, 18 in *Brachypodium*, 22 in rice, 25 in *Brassica rapa*, and 40 in wheat; Melo et al., 2020), which are divided into many subfamilies. Expression levels of the SCL30a, SCL28, and SCL33 genes were altered upon treatment with ABA (Cruz et al., 2014), indicating that SR proteins respond to ABA. Zhang et al. (2014) noticed that splicing factor SR34b mutation reduces cadmium tolerance in *Arabidopsis* by regulating iron-regulated transporter 1 gene. Besides its response to high light intensities (Tanabe et al., 2008), *Arabidopsis* SR45 participates as a suppressor to innate immunity (Zhang et al., 2017). Likewise, SR protein RSZ21 obtained from *Arachis* has been found to play a role in plant defense and HR-like cell death (Kumar and Kirti, 2012). Yoon et al. (2018) discovered altered expression levels of 78.6% genes (22 out of 28) and 60.7% of AS patterns in *Brassica rapa* in response to abiotic stresses. The highest expressions were detected when plants were exposed to oxidative, cold, and heat treatments. Interestingly, cold and heat stresses caused the most AS events. These studies point out that type of abiotic stress largely influences the expression patterns of SR proteins and also the AS patterns. Using CRISPR/Cas9-mediated plant genome engineering, Butt et al. (2019) targeted each rice SR locus and produced single knockouts. Such a study is extremely vital and forms a useful resource material to understand the role of SR proteins in plant development as well as abiotic stresses.

## Do ncRNAs Have a Role in the Regulation of Lys and Ser Pathways and its Interactions?

Major part of the genome is non-coding and is transcribed into non-coding RNAs (ncRNAs) only, which is known to play a regulatory role. Among these ncRNAs, long non-coding RNAs (lncRNAs) are known to be involved in regulation of gene expression. However, very little is known about the role of ncRNAs in providing tolerance against biotic stress including plant diseases. The interactions of lncRNAs with proteins in plants using a system genomic approach is promising and is relevant to ascertain characteristic trait biology relationships. In the recent past, several databases for genes encoding lncRNAs in plants have become available, which include greeNC (Gallart et al., 2016), CantataDB (Szczesniak et al., 2016), PLncDB (Jin et al., 2013) etc. However, these databases lack *bona fide* entries and have poor annotations. In plants, lncRNAs are known to perform multiple biological functions, which include the following: phosphate homeostasis (Bazin and Bailey-Serres, 2015), flowering (Swiezewski et al., 2009), photomorphogenesis (Wang et al., 2014), stress response (Yuan et al., 2018), fertility (Liu et al., 2017), etc. The lncRNAs are also known to function as target mimics of miRNAs (Aung et al., 2006; Pant et al., 2008) and regulate post-translational processes *via* protein modifications and protein-protein interactions (PPIs; Liu et al., 2015), etc. Song and Zhang (2017) have extensively reviewed the characteristics, identification, and functions of lncRNAs in response to various stresses. The authors delve into a greater understanding and need of nutrient deficiency and the factors associated with abiotic stress. We also argue that the identification of regulatory elements associated with a disease and validating them through NGS has been a routine task. In this process, distinct signatures in the form of lncRNAs in plants would be ideal candidates for studying agronomically important traits. It is a challenge to identify ncRNAs that were not characterized earlier and to find if they are specific to any trait/genotype. For example, the miRNA target analysis also divulged that *DHNs* are targeted by 51 miRNAs responsive to abiotic stress. The gene expressions are common and well-coordinated under diverse abiotic stress conditions and *DHNs* are no exception. The role of DHNs under different abiotic stress conditions, the regulatory networks of *DHN* genes, and their physiological functions have been discussed (Yu et al., 2018). In *S. bicolor*, transcript expressions were higher in roots, stems, and leaves in comparison with inflorescences (Nagaraju et al., 2018). While all *DHN* genes exhibited high expressions in stems under cold, heat, salt, and drought stresses, *SbDHN2* displayed the highest expression under multiple stresses in all the tissues of *S. bicolor* (Nagaraju et al., 2018). These results infer that the involvement of SbDHN2 of YnS group in a wide array of stresses. In the recent past, studies on proteome diversity revealed that lncRNAs play a very important role in serine/arginine (SR) regulations (Fesenko et al., 2017). Furthermore, the role of lncRNAs in nonsense-mediated mRNA decay was identified under differential alternative splicing in relation to altitude, not the abiotic stress, which is beyond the scope of this review. Furthermore, Ser and Lys metabolism together are not explicitly known.

## Conclusions and Outlook

Both Lys and Ser are key amino acids, involved in plant ontogeny and also connected with abiotic stress tolerance. The biosynthetic pathways are multiple and complex. Among the pathways that exist, the PPSB appears to play a critical role during growth and development. Diverse mechanisms associated with biosynthesis and catabolism of these two important amino acids however, need to be elucidated and also their interaction with other pathways. Attempts were made to enrich the seeds of rice and others with Lys, but still problems exist with regard to seed morphology. These shortcomings need to be overcome in future in order to make high Lys-containing lines available to the public. Further, it is concluded that Lys is catabolized to serve as an alternative respiratory substrate since photosynthetic activity is impaired under abiotic stress conditions. Thus, plants get detoxified by supplementing the much-needed energy during the time of abiotic stress recovery. This vital function of Lys is in addition to its regular participation in protein synthesis and other metabolic activities. Such functions to the amino acids are novel and were not predicted earlier. Many functions of Lys and Ser during growth and development have come into light recently, though the mechanistic explanations are not clear. Further, Lys- and Ser-rich proteins and their induction during abiotic stress reveals the crucial role they play in a wide spectrum of abiotic stresses. Identification of the *cis*- and *trans*-acting elements associated with the activation/suppression of the biosynthetic pathway genes and subsequent events that take place in the downstream remains elucidated. Finally, lncRNAs appear to play a role in these processes by interacting with serine kinase and several novel proteins. Mapping those miRNAs and lncRNAs, their interaction and the key roles they play during the regulation of these processes is certainly the biggest challenge for future research.

## Author Contributions

PBK wrote the first draft with sections on Serine and split by all other authors. RS developed the figures. PS wrote sections on lncRNAs. PBK, PS, and RP proofread the manuscript before all the agreeing to submission. All authors contributed to the revisions and approved the submitted version.

### Conflict of Interest

The authors declare that the research was conducted in the absence of any commercial or financial relationships that could be construed as a potential conflict of interest.

## Footnotes

^1^http://www.arabidopsis.org/
